# BMP-2 Variants in Breast Epithelial to Mesenchymal Transition and Microcalcifications Origin

**DOI:** 10.3390/cells9061381

**Published:** 2020-06-02

**Authors:** Manuel Scimeca, Raffaella Giocondo, Manuela Montanaro, Annarita Granaglia, Rita Bonfiglio, Virginia Tancredi, Alessandro Mauriello, Nicoletta Urbano, Orazio Schillaci, Elena Bonanno

**Affiliations:** 1 Department of Biomedicine and Prevention, University of Rome “Tor Vergata”, Via Montpellier 1, 00133 Rome, Italy; manuel.scimeca@uniroma2.it (M.S.); orazio.schillaci@uniroma2.it (O.S.); 2 San Raffaele University, Via di Val Cannuta 247, 00166 Rome, Italy; 3 Saint Camillus International University of Health Sciences, Via di Sant’Alessandro, 8, 00131 Rome, Italy; 4 Department of Systems Medicine, University of Rome “Tor Vergata”, Via Montpellier 1, 00133 Rome, Italy; raffaellagiocondo@gmail.com (R.G.); tancredi@uniroma2.it (V.T.); 5 Department of Experimental Medicine, University of Rome “Tor Vergata”, Via Montpellier 1, 00133 Rome, Italy; manuelamontanaro1991@gmail.com (M.M.); bonfiglio.rita@gmail.com (R.B.); alessandro.mauriello@uniroma2.it (A.M.); 6 ’Diagnostica Medica’ and ‘Villa dei Platani’, Neuromed Group, 83100 Avellino, Italy; a.granaglia@gmail.com; 7 Fondazione Umberto Veronesi (FUV), Piazza Velasca 5, 20122 Milano, Italy; 8 Centre of Space Biomedicine, University of Rome “Tor Vergata”, Via Montpellier 1, 00133 Rome, Italy; 9 Tor Vergata Oncoscience Research (TOR), University of Rome “Tor Vergata”, 00133 Rome, Italy; 10 Nuclear Medicine, Policlinico “Tor Vergata”, viale Oxford, 81, 00133 Rome, Italy; n.urbano@virgilio.it; 11 IRCCS Neuromed, Via Atinense, 18, 86077 Pozzilli, Italy

**Keywords:** breast cancer, BMP-2, epithelial to mesenchymal transition, microcalcifications, breast osteoblast-like cells (BOLCs)

## Abstract

This study aims to investigate the possible different roles of the BMP-2 variants, cytoplasmic and nuclear variant, in both epithelial to mesenchymal transition and in microcalcifications origin in human breast cancers. To this end, the in situ expression of cytoplasmic and nuclear BMP-2 was associated with the expression of the main epithelial to mesenchymal transition biomarkers (e-cadherin and vimentin) and molecules involved in bone metabolisms (RUNX2, RANKL, SDF-1) by immunohistochemistry. In addition, the expression of cytoplasmic and nuclear BMP-2 was associated with the presence of microcalcifications. Our data showed a significant association among the number of cytoplasmic BMP-2-positive cells and the number of both vimentin (positive association) and e-cadherin (negative association) positive breast cells. Conversely, no associations were found concerning the nuclear BMP-2-positive breast cells. Surprisingly, the opposite result was obtained by analyzing the variants of BMP-2 and both the expression of RANKL and SDF-1 and the presence of microcalcifications. Specifically, the presence of microcalcifications was related to the expression of nuclear BMP-2 variant rather than the cytoplasmic one, as well as a strong association between the number of nuclear BMP-2 and the expression of the main breast osteoblast-like cells (BOLCs) biomarkers. To further corroborate these data, an in vitro experiment for demonstrating the co-expression of nBMP-2 and RANKL or vimentin or SDF-1 in breast cancer cells that acquire the capability to produce microcalcifications was developed. These investigations confirmed the association between the nBMP-2 expression and both RANKL and SDF-1. The data supports the idea that whilst cytoplasmic BMP-2 can be involved in epithelial to mesenchymal transition phenomenon, the nuclear variant is related to the essential mechanisms for the formation of breast microcalcifications. In conclusion, from these experimental and translational perspectives, the complexity of BMP-2 signaling will require a detailed understanding of the involvement of specific BMP-2 variants in breast cancers.

## 1. Introduction

Breast cancer is one of the most common female neoplasia that causes high morbidity and mortality worldwide. In Europe, a total of 404,920 new female breast cancer cases were estimated to have occurred in 2018, with 138,000 deaths [1]. In recent years, several molecular mechanisms involved in breast cancer occurrence and development have been described. Among them, the epithelial to mesenchymal transition (EMT) phenomenon is considered one of the most important mechanisms related to the development of metastatic lesions in breast cancer [2,3]. The EMT is morphologically characterized by the loss of epithelial phenotype and the acquisition of a mesenchymal aspects through the decreased expression of e-cadherin and increased expression of vimentin and n-cadherin [2,3]. The EMT is morphologically characterized by the loss of epithelial phenotype through the decreased expression of e-cadherin and the acquisition of a mesenchymal phenotype, mainly by increasing the expression of vimentin and n-cadherin [2,3]. Scimeca et al. recently demonstrated that the EMT process was linked to the transformation of breast cancer cells into cells with osteoblast-like characteristics via bone morphogenetic proteins (BMP)-2 stimulation [4]. The authors also demonstrated that these cells, the breast osteoblast-like cells (BOLCs), were capable of producing calcium minerals with a mechanism similar to the formation of hydroxyapatite in bone. In addition, a recent study displayed a correlation between the presence of BOLCs and the development of bone metastatic lesions within five years from histological diagnosis [4]. It is known that BOLCs express typical biomarkers of bone cells, such as BMP-2, BMP-4, osteopontin (OPN) and pentraxin-related protein 3 (PTX3) [4]. However, some studies suggest that these molecules can also be directly involved in the EMT phenomenon in breast cancer. In particular, Huang et al. demonstrated that the expression of BMP-2 may facilitate the occurrence of EMT and bone metastasis in breast cancer xenograft models [5]. To complicate the understanding of the role of BMP-2 in human breast cancers, biological function studies have explained the dual role of Bone Morphogenetic Proteins (BMPs) in both cancer development and suppression. In addition, a variety of antagonists of BMP molecules, ligands, and receptors have been shown to reduce or enhance tumorigenesis and metastasis. A nuclear variant of BMP-2 has been recently identified. Specifically, nBMP-2 is the nuclear variant of the BMP-2, produced by translation from an alternative downstream start codon that, eliminating the N-terminal endoplasmic reticulum signal peptide, prevents the N-terminal endoplasmic reticulum signal peptide. Further, nBMP-2 is translated in the cytoplasm and translocated to the nucleus by means of an embedded bipartite nuclear localization signal. Freitas et al. investigated the possible role of nBMP-2 macrophages isolated from naïve (uninfected) nBmp2NLS^tm^ mutant mice, demonstrating that the impairment of nBMP-2 induces alteration in intracellular Ca^2+^ transport [6]. This datum was in agreement with the evidence that the impairment of nBMP-2 leads to muscular, neurological, and immune phenotypes, all of which are consistent with aberrant intracellular calcium (Ca^2+^) response in a mouse model.

Starting from these considerations, this study aims to investigate the possible different roles of cytoplasmic (here identify as BMP-2) and nuclear (here identify as nBMP-2) BMP-2 in both the EMT and microcalcifications production in human breast cancer. To this end, the in situ expression of BMP-2 and nBMP-2 in human breast cancer was correlated with the expression of the main EMT biomarkers (e-cadherin and vimentin) and molecules involved in bone metabolisms (runt-related transcription factor 2, RUNX2; receptor activator of nuclear factor kappa-Β ligand, RANKL; stromal cell-derived factor, SDF-1). In addition, an in vitro experiment for demonstrating the co-expression of nBMP-2 and RANKL or vimentin or SDF-1 in breast cancer cells that acquire the capability to produce microcalcifications was developed.

## 2. Materials and Methods

From December 2018 to December 2019, we collected 150 breast samples (age, 56.57 ± 13.77 y; range, 26 to 88 y). From each biopsy, paraffin serial sections were obtained to perform histological classification and immunohistochemical analysis. In addition, 1 mm^3^ tissue fragments were used to perform microanalytical (EDX-microanalysis) investigations. The study protocol was approved by the “Policlinico Tor Vergata” Independent Ethical Committee (reference number # 129.18, 26 July 2018). Written patient consent was obtained for each patient.

### 2.1. Histology

After fixation in 10% buffered formalin for 24 h, the breast tissues were embedded in paraffin. Three-micrometer-thick sections were stained with hematoxylin and eosin (H & E), and the diagnostic classification was blindly performed by two pathologists [7].

### 2.2. Immunohistochemistry

Immunohistochemical analyses were performed in order to investigate the expression of BMP-2, vimentin, e-cadherin, runt-related transcription factor 2 (RUNX2), receptor activator of nuclear factor kappa-Β ligand (RANKL) and stromal cell-derived factor (SDF)-1 in breast tissues. Briefly, antigen retrieval was performed on 3-μm-thick paraffin sections using EDTA citrate pH 7.8 or citrate pH 6.0 buffers for 30 min at 95 °C. Sections were then incubated for 1 h at room temperature with the primary antibodies reported in the Table 1. Washings were performed with PBS/Tween20 pH 7.6. Reactions were revealed by the HRP-DAB Detection Kit (UCS Diagnostic, Rome, Italy). For each marker, immunohistochemical reactions were evaluated by counting the number of positive breast cells on 500 in total in randomly selected regions. Concerning the evaluation of BMP-2, for each sample, we counted the number of both cytoplasmic and nBMP-2-positive cells on a total of 500 randomly selected regions.

### 2.3. Transmission Electron Microscopy (TEM) of Breast Tissues

One millimeter^3^ of breast tissue from each surgical specimen was fixed in 4% paraformaldehyde (PFA) and post-fixed in 2% osmium tetroxide [8]. After washing with 0.1 M phosphate buffer, the sample was dehydrated by a series of incubations in 30%, 50%, and 70% ethanol. Dehydration was continued by incubation steps in 95% ethanol, absolute ethanol, and propylene oxide; then, samples were embedded in EPON (Agar Scientific, Stansted Essex CM24 8GF United Kingdom) [8]. Eighty µm ultra-thin sections were mounted on copper grids and observed with Hitachi 7100FA transmission electron microscope (Hitachi, Schaumburg, IL, USA).

### 2.4. Energy Dispersive X-ray (EDX) Microanalysis

All breast samples underwent energy dispersive X-ray (EDX) microanalysis. Six-micrometer-thick paraffin sections were embedded in EPON resin as previously described by the authors of [9], followed by the identification of microcalcifications. Briefly, sections were deparaffinized, hydrated, osmium tetroxide-fixed, dehydrated in ethanol and propylene oxide, and infiltrated in EPON. The embedding capsules were positioned over areas containing previously identified microcalcifications. Unstained ultra-thin sections of approximately 100 nm thickness were mounted on copper grids for microanalysis. EDX spectra of microcalcifications were acquired with a Hitachi 7100FA transmission electron microscope (Hitachi, Schaumburg, IL, USA) and an EDX detector (Thermo Scientific, Waltham, MA, USA) at an acceleration voltage of 75 KeV and magnification of 12,000. Spectra were semi-quantitatively analyzed by the Noran System Six software (Thermo Scientific, Waltham, MA, USA) using the standardless Cliff–Lorimer k-factor method [10]. The EDX microanalysis apparatus was calibrated using an X-ray microanalysis standard (Micro-Analysis Consultants Ltd., Cambridgeshire, UK).

### 2.5. Calcium Oxalate Synthesis

The CO was synthesized as follows. Seven L of distilled water was placed in a crystallizer and heated to 70 °C. Solutions of Na_2_C_2_O_4_ (7.5 × 10^−3^ M) and CaCl_2_ (7.5 × 10^−3^ M) were dropped simultaneously at the same speed (250 mL per h). The slurry was filtered, and the crystals were washed with water and ethanol and then dried at 50 °C with a vacuum for 24 h.

### 2.6. Cell Culture

The BT-474 cells were obtained from the American Type Culture Collection (ATCC; Manassas, VA, USA) and maintained by the Cell and Tissue Culture Core, Lombardi Cancer Center (Washington DC, USA). The cells were routinely cultured in DMEM high glucose (Sigma-Aldrich, St Louis, MO, USA) supplemented with 10% fetal bovine serum (FBS).

### 2.7. In Vitro Model for the Development of “Osteoblast-Like Cells”

For each experiment, about 10,000 BT-474 were cultured. According to our previous study, in which we demonstrated the ability of CO to induce osteoblast differentiation of breast cancer cell lines [x], the experimental scheme was BT-474 + calcium oxalate (BT-474/CO) and BT-474 + hydroxyapatite (BT-474/HAP) and BT-474 alone (BT-474/CTRL) as controls. After 10–12 days, the cells were used for subsequent experiments.

### 2.8. Cell Culture Immunoflurescence

The cells were plated on poly-l-lysine coated slides (Sigma-Aldrich cat #P4707) in 24-well cell culture plates and fixed in 4% paraformaldehyde. After pre-treatment with EDTA citrate at 95 °C for 20 min and 0.1% Triton X-100 for 15 min, the cells were incubated 1 h with the mouse monoclonal anti-BMP-2 antibody and the rabbit monoclonal anti-RANKL antibody or the mouse monoclonal anti-vimentin antibody or the mouse monoclonal anti-SDF-1 antibody (see Table 1). Washings were performed with PBS/Tween20 pH 7.6. Reactions were revealed by using FITC-goat anti-mouse secondary antibodies (Novus Biologicals, Littleton, CO, USA) for BMP-2, Texas red-goat anti-rabbit secondary antibodies (Novus Biologicals, Littleton, CO, USA) for RANKL, and Texas red-goat anti-mouse secondary antibodies (Novus Biologicals, Littleton, CO, USA) for both vimentin and SDF-1. Lastly, DAPI (Novus Biologicals, Littleton, CO, USA) was used to stain the nucleus. RANKL and nBMP-2 co-expression were evaluated by counting the number of RANKL-nBMP-2-positive breast cancer cells out of a total of 100 in randomly selected regions.

### 2.9. Statistical Analysis

Statistical analysis was performed by using GraphPad Prism 5 Software (San Diego, CA, USA). The analysis of nBMP-2/BMP-2 expression in breast cancers, as well as the comparison between nBMP-2 expression and the type of breast microcalcifications, were performed by the Kruskal–Wallis test (*p* < 0.05) and by the Mann–Whitney test (*p* < 0.05). Linear regression analyses were performed to assess the association among BMP-2 and nBMP-2 and the expression of vimentin, e-cadherin, RUNX2, RANKL, and SDF-1. For the in vitro data, analyses of the number of immunofluorescence-positive BT-474 cells were performed by the Kruskal–Wallis test (*p* < 0.05) and by the Mann–Whitney test (*p* < 0.05).

## 3. Results

### 3.1. Morphological Classification of Breast Lesions

In agreement with the WHO [11], breast samples were classified as follows: 48 benign lesions (24 fibrocystic mastopathies, 19 fibroadenomas, and 5 flat-atypia) and 102 breast malignant lesions (2 in situ comedonic carcinomas, 34 in situ ductal carcinomas, and 66 infiltrating ductal carcinomas—40 G1 grade, 16 G2 grade, and 10 G3 grade). Concerning the benignant lesions, microcalcifications were found in 20 samples (2 fibrocystic mastopathies, 14 fibroadenomas, and 4 flat-atypia). In the malignant lesions, 54 samples were positive for the research of microcalcifications (2 in situ comedonic carcinomas, 25 in situ ductal carcinomas, and 27 infiltrating ductal carcinomas—17 G1 grade, 6 G2 grade, 4 G3 grade).

### 3.2. Analysis of nBMP-2/BMP-2 Expression in Breast Cancers

**Figure 1:**
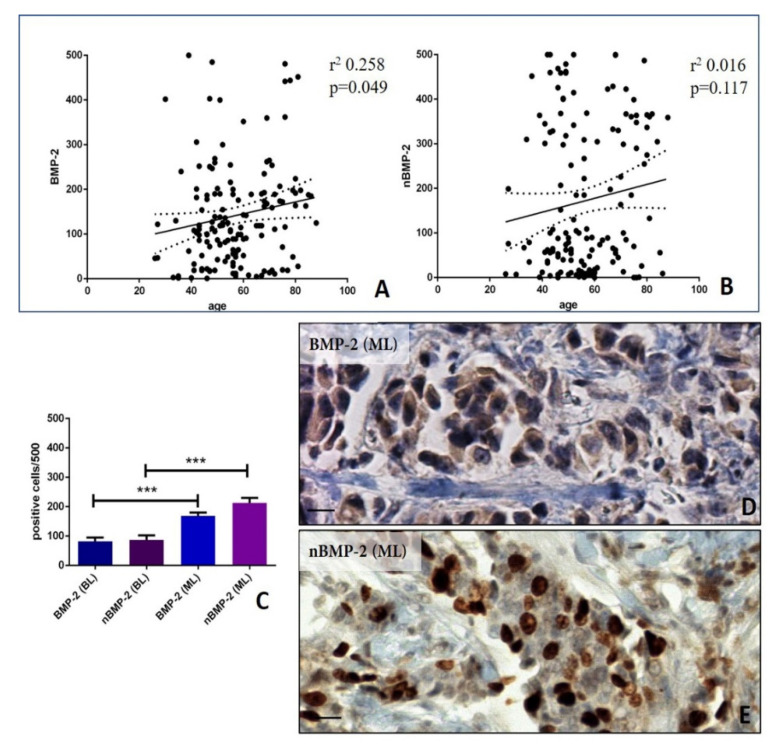
Cytoplasmic and nuclear expression of BMP-2 in breast tissues. (**A**) The graph shows a significant association between the number of BMP-2-positive cells and patients’ age. (**B**) The graph displays a significant association between the number of nBMP-2-positive cells and patients’ age. (**C**) The graph shows the number of positive BMP-2 and nBMP-2 in breast benign and malignant lesions. (**D**) The infiltrating breast carcinomas with numerous cytoplasmic BMP-2-positive cells. (**E**) The infiltrating breast carcinomas with numerous nBMP-2-positive cells. The scale bar represents 200 µm for each image. (* *p* ≤ 0.05, ** *p* ≤ 0.01, *** *p* ≤ 0.001, **** *p* ≤ 0.0001).

In order to investigate the different role of nBMP-2 and BMP-2 in breast cancer progression, the expression of these molecules has been compared to age and evaluated in benign and malignant lesions. Concerning the linear regression analysis, a significant association was observed between the number of BMP-2-positive cells and the patients’ age, regardless of the lesion type (r^2^ 0.258; *p* = 0.049) (Figure 1A). Conversely, no significant association was found considering the nBMP-2-positive cells (r^2^ 0.016; *p* = 0.117) (Figure 1B).

A significant increase in the number of both BMP-2- (BL 82.00 ± 12.64; ML 168.9 ± 212.8) and nBMP-2 (BL 87.42 ± 15.21)-positive breast cells was observed in malignant lesions as compared to benignant ones (BMP-2 *p* < 0.0001; nBMP-2 *p* < 0.0001) (Figure 1C–E). No differences were observed concerning the number of BMP-2- and nBMP-2-positive breast cancer cells in the malignant group (*p* = 0.478) (Figure 1C).

### 3.3. BMP-2/nBMP-2 and the Epithelial to Mesenchymal Transition

**Figure 2:**
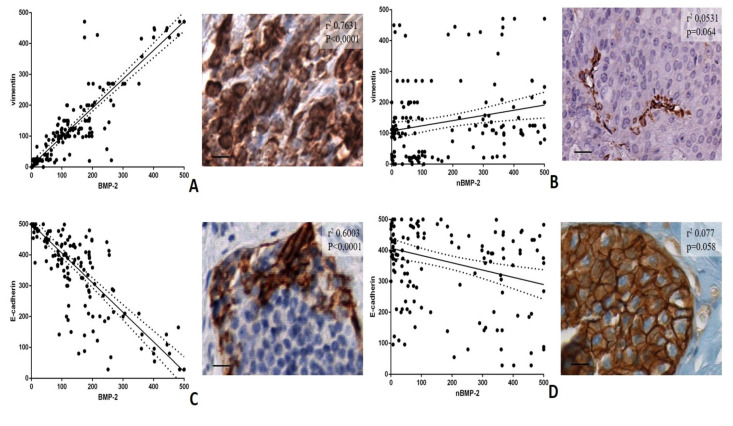
Linear regression analysis of the association among cytoplasmic BMP-2, nBMP-2, and EMT markers vimentin and e-cadherin. (**A**) The graph shows a significant positive association between the number of BMP-2-positive cells and vimentin. The image displays numerous vimentin-positive breast cancer cells in a case of infiltrating carcinomas (**B**) The graph displays no association between the number of nBMP-2-positive cells and vimentin. The image shows rare vimentin-positive breast cancer cells in a case of infiltrating carcinomas. (**C**) The graph shows a significant inverse association between the number of BMP-2-positive cells and e-cadherin. The image displays numerous e-cadherin negative breast cancer cells. (**D**) The graph displays no association between the number of nBMP-2-positive cells and e-cadherin. The image shows e-cadherin-positive breast cancer cells. The scale bar represents 200 µm for each image.

Linear regression analyses were performed to evaluate the possible association among BMP-2 and nBMP-2 and the main markers of EMT, such as vimentin and e-cadherin (Figure 2A–D).

As concerns the analysis of BMP-2, significant associations were found for both vimentin (r^2^ 0.7631; *p* < 0.0001) and e-cadherin (r^2^ 0.6003; *p* < 0.0001) (Figure 2A,C). Surprisingly, no significant association was observed between nBMP-2 and both vimentin (r^2^ 0.0531; *p* = 0.064) and e-cadherin (r^2^ 0.077; *p* = 0.058) (Figure 2B,D). The infiltrating breast carcinomas characterized by a higher number of vimentin-positive breast cancer cells frequently showed no or rare positivity for nBMP-2. Therefore, the EMT phenomenon in breast cancer seems to be related to the expression of BMP-2 rather than nBMP-2.

### 3.4. BMP-2/nBMP-2 and the Formation of Breast Microcalcifications

The presence of breast microcalcifications was investigated in both the benign and malignant lesions to study the possible association between the nBMP-2 expression and calcifications formation.

**Figure 3:**
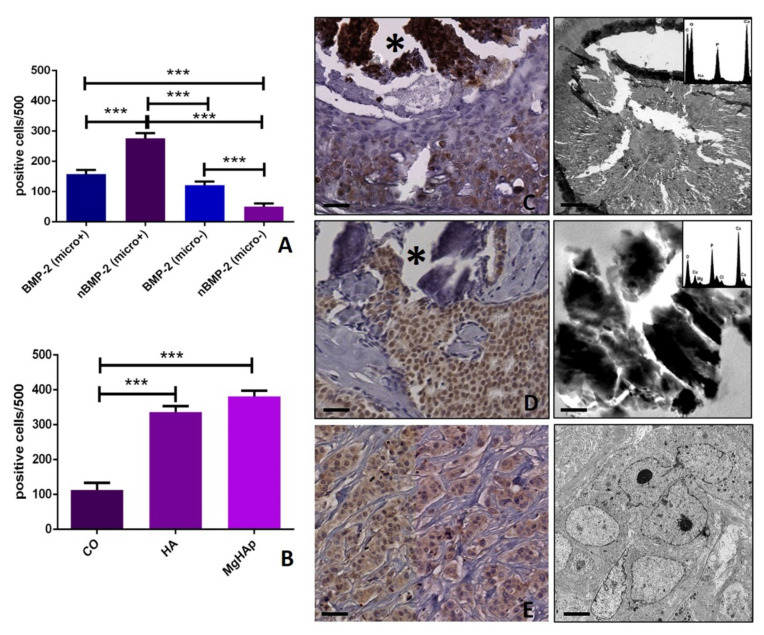
Cytoplasmic and nuclear BMP-2 in breast microcalcifications. (**A**) The graph displays the expression of cytoplasmic and nuclear BMP-2 in breast tissues with or without microcalcifications. (**B**) The graph shows the expression of nuclear BMP-2 in breast tissues with calcifications made of calcium oxalate (CO), hydroxyapatite (HA) or magnesium-substituted hydroxyapatite (Mg–HAp). The elemental composition of microcalcification has been performed on a paraffin serial section by transmission electron microscope and energy dispersive X-ray microanalysis apparatus. (**C**) The image shows some cytoplasmic BMP-2-positive breast cells in an infiltrating breast carcinoma next to a microcalcification (the asterisk). The transmission electron microscope and energy dispersive X-ray microanalysis show that microcalcification is made of HA (spectrum). (**D**) Numerous nBMP-2-positive cells in an infiltrating breast carcinoma close to a microcalcification (the asterisk). The transmission electron microscope and energy dispersive X-ray microanalysis show that microcalcification is made of Mg–HAp. (**E**) Cytoplasmic and nuclear BMP-2-positive breast cells in an infiltrating breast carcinoma without microcalcifications. The electron micrograph shows a cluster of infiltrating breast cancer cells. The scale bar represents 100 µm for each immunohistochemical image. Transmission electron microscope scale bars: A, 0.2 µm; B, 0.2 µm; C, 5 µm. (* *p* ≤ 0.05, ** *p* ≤ 0.01, *** *p* ≤ 0.001, **** *p* ≤ 0.0001).

Specifically, to evaluate the possible different role of BMP-2 and nBMP-2 in breast microcalcifications production, their expression was evaluated in all collected samples (*p* < 0.0001). Surprisingly, a very significant difference was observed concerning the expression of BMP-2 (micro+ 157.0 ± 13.25; micro− 121.9 ± 12.38) and nBMP-2 (micro+ 273.2 ± 16.60; micro− 51.40 ± 10.20) in breast lesions with or without calcifications (micro+ BMP-2 vs. nBMP-2 *p* < 0.0001; micro− BMP-2 vs. nBMP-2 *p* < 0.0001) (Figure 3A–E). In particular, the presence of microcalcifications seems to be related to the expression of nBMP-2 rather than BMP-2 (Figure 3A).

The capability of nBMP-2 expression to predict the type/elemental composition of microcalcifications was investigated by correlating the number of positive nBMP-2 and the presence of calcium oxalate (CO), hydroxyapatite (HA) and magnesium-substitute hydroxyapatite (Mg–HAp) (Figure 3B–E). The elemental composition of breast microcalcifications was previously investigated on a paraffin serial section by using the TEM and EDX apparatus. A significant increase in the number of nBMP-2-positive cells was found in both HA (335.9 ± 17.19) and Mg–HAp (381.5 ± 15.77) groups if compared with lesions characterized by the presence of CO (112.7 ± 20.4) (CO vs. HA *p* < 0.0001; CO vs. Mg–HAp *p* < 0.0001; HA vs. Mg–HAp *p* = 0.0966). Also, a significant group effect was found (*p* < 0.0001) (Figure 3B).

**Figure 4:**
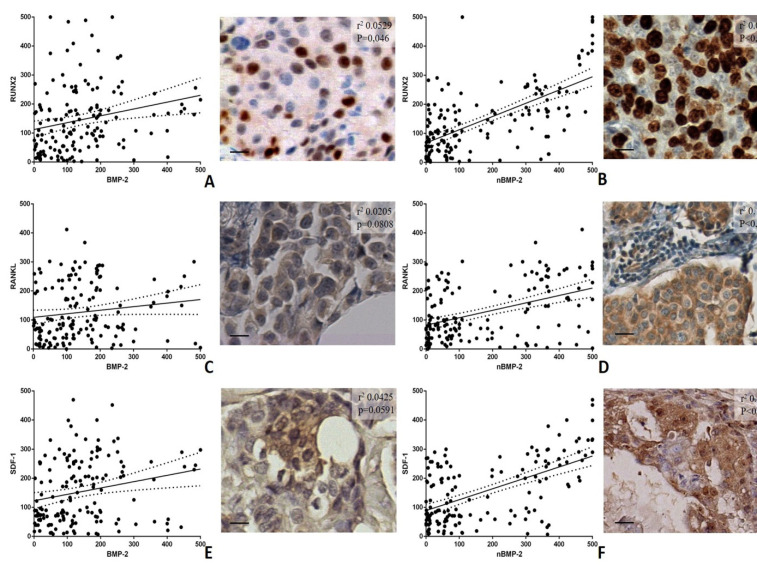
Linear regression analysis of the association among cytoplasmic BMP-2, nBMP-2, and bone biomarkers RUNX2, RANKL and SDF-1. (**A**) The graph displays significant association between the number of BMP-2-positive cells and RUNX2. The image shows some RUNX2-positive breast cancer cells in an infiltrating breast carcinoma. (**B**) The graph shows a significant association between the number of nBMP-2-positive cells and RUNX2. The image displays numerous RUNX2-positive breast cancer cells in an infiltrating breast carcinoma. (**C**) The graph displays no association between the number of BMP-2-positive cells and RANKL. The image shows some RANKL-positive breast cancer cells in an infiltrating breast carcinoma. (**D**) The graph displays significant association between the number of nBMP-2-positive cells and RANKL. The image displays several RANKL-positive breast cancer cells in an infiltrating breast carcinoma. (**E**) The graph displays no association between the number of BMP-2-positive cells and SDF-1. The image shows rare SDF-1-positive breast cancer cells in an infiltrating breast carcinoma. (**F**) The graph shows significant association between the number of nBMP-2-positive cells and SDF-1. The image shows some SDF-1-positive breast cancer cells in an infiltrating breast carcinoma. The scale bar represents 200 µm for each image.

To further corroborate this data, linear regression analyses of the association among BMP-2 and nBMP-2 and the main markers of the cells involved in microcalcifications production (the BOLCs) were carried out. In particular, both nBMP-2 and BMP-2 were associated with the expression of the following BOLCs biomarkers: RUNX2, RANKL, and SDF-1 (Figure 4).

For RUNX2, a significant association was observed for both BMP-2 (r^2^ 0.0529; *p* = 0.0046) and nBMP-2 (r^2^ 0.00014; *p* < 0.0001) (Figure 4A,B). However, as seen in Figure 4, nBMP-2 showed a higher association with RUNX2 if compared to BMP-2 (Figure 4A,B). The analyses of markers of mature osteoblasts, RANKL, and SDF-1, showed significant association with nBMP-2 (RANKL r^2^ 0.1919; *p* < 0.0001; SDF-1 r^2^ 0.3193; *p* < 0.0001) but not with BMP-2 (RANKL r^2^ 0.0205; *p* = 0.0808; SDF-1 r^2^ 0.0425; *p* = 0.0591) (Figure 4C–F). Therefore, nBMP-2 displayed a higher association with the expression of biomarkers of the mature BOLCs involved in the production of breast microcalcifications than BMP-2 (Figure 4C–F).

### 3.5. In Vitro Model

According to our previous study, in which we demonstrated the ability of CO to induce osteoblast differentiation of breast cancer cell lines [12], we performed in vitro investigations in order to confirm the association between nBMP-2 expression and the formation of breast microcalcifications. To this end, morphological and immunofluorescence analysis were made.

#### Immunofluorescence Analysis

**Figure 5:**
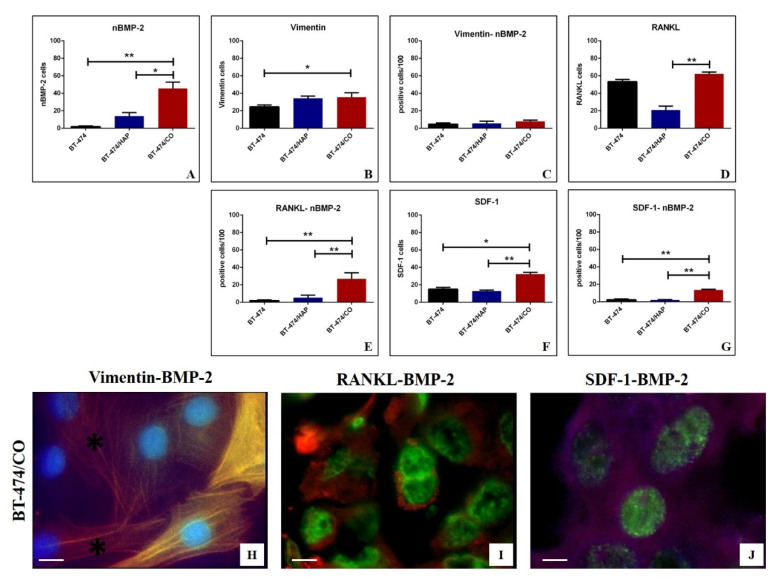
In vitro analysis of BMP-2 and RANKL expression in BT-474 breast cancer cells showing an osteoblast-like phenotype (**A**) The graph displays the number of nBMP-2-positive breast cells among the BT-474, BT-474/HAP, and BT-474/CO groups. (**B**) The graph shows the number of vimentin-positive breast cells among the BT-474, BT-474/HAP, and BT-474/CO groups. (**C**) The graph displays the number of vimentin-nBMP-2-positive breast cells among the BT-474, BT-474/HAP, and BT-474/CO groups. (**D**) The graph shows the number of RANKL-positive breast cells among the BT-474, BT-474/HAP, and BT-474/CO groups. (**E**) The graph displays the number of RANKL-nBMP-2-positive breast cells among the BT-474, BT-474/HAP, and BT-474/CO groups. The graph displays several cytoplasmatic BMP-2-positive cells in the BT-474/HAP group. (**F**) The graph shows the number of SDF-1-positive breast cells among the BT-474, BT-474/HAP, and BT-474/CO groups. (**G**) The graph displays the number of SDF-1- nBMP-2-positive breast cells among the BT-474, BT-474/HAP, and BT-474/CO groups. (**H**) A representative image of the BT-474/CO group in which breast cancer cells express only vimentin (red; asterisks) or both vimentin and cytoplasmic BMP-2 (green). (**I**) The image shows numerous RANKL- (red) nBMP-2 (green)-positive breast cancer cells in the BT-474/CO group. (**J**) The image displays numerous SDF-1- (red) nBMP-2 (green)-positive breast cancer cells in the BT-474/CO group. The scale bar represents 10 µm for each immunofluorescent image. (* *p* ≤ 0.05, ** *p* ≤ 0.01, *** *p* ≤ 0.001, **** *p* ≤ 0.0001).

Immunofluorescence analysis showed a significant group effect of the nBMP-2 expression among experimental groups (*p* = 0.048) (Figure 5A). Concerning the post-hoc test, a significant increase in the number of nBMP-2-positive breast cancer cells was displayed in BT-474/CO as compared to both BT-474/CTRL (*p* = 0.022) and BT-474/HAP (*p* = 0.009) (Figure 5A). Concerning the number of vimentin-positive breast cancer cells, a significant group effect was observed (*p* = 0.041). In addition, we noted a significant increase in the number of vimentin-positive breast cancer cells in BT-474/CO group as compared to BT-474/CTRL one (*p* = 0.044) (Figure 5B). Interestingly, no significant difference was observed considering the co-expression of vimentin and nBMP-2 (Figure 5C). The analysis of the RANKL expression among BT-474/CTRL, BT-474/CO and BT-474/HAP showed a significant group effect (*p* = 0.0036) (Figure 5D). The post-hoc test displayed a significant increase in the number of RANKL-positive cells in BT-474/CO as compared to BT-474/HAP (*p* = 0.039) (Figure 5D). Similarly, in the presence of CO (BT-474/CO) a significant increase in the number of RANKL-nBMP-2-positive breast cancer cells was observed when compared to both the BT-474/CTRL (*p* = 0.022) and BT-474/HAP (*p* = 0.009) groups (Figure 5E). A similar trend was also observed studying both the expression of SDF-1 alone (Figure 5F) or with nBMP-2 (Figure 5G). Of interest, we frequently observed both vimentin-positive breast cancer cells and vimentin-cytoplasmic BMP-2 co-positive cells in the BT-474/CTRL group (Figure 5H). In the same group, cells showing an osteoblast-like phenotype frequently co-expressed RANKL and nBMP-2 or and SDF-1 and nBMP-2 (Figure 5I,J). No significant differences were observed concerning the co-expression of RUNX2 and nBMP-2 among the experimental groups (data not shown).

## 4. Discussion

Recently, the occurrence of the EMT phenomenon in the breast has been associated with the origin of BOLCs and the resulting formation of breast microcalcifications made of HA or Mg–HAp [12,13,14,15,16]. However, historically, EMT is considered a biological mechanism related to breast cancer development and metastasis formation [17,18]. Notably, the evidence that the presence of BOLCs increases the risk of bone metastasis development in breast cancer patients [4] can ideally connect these two sides of the same coin. Indeed, both the EMT occurrence and the presence of BOLCs are linked to the breast cancer metastatization. There are several molecules capable of inducing the EMT phenomenon in breast cancer [19]. Among them, several studies propose the BMPs as EMT-inducing factors [20]. These molecules are generally involved in bone metabolism as well as the formation of ectopic calcifications [21]. Thus, it is possible that in breast cancer, BMPs, and in particular BMP-2, can be involved simultaneously in EMT, BOLCs origin, and the formation of microcalcifications. To further complicate matters, Tellez Freitas et al. recently demonstrated that the nuclear variant of BMP-2 (nBMP2) is expressed in macrophages and can alter the calcium homeostasis [6]. Therefore, it is possible to hypothesize that the occurrence of these two events is due to the presence of the two variants of BMP-2.

Starting from these considerations, this study aimed to investigate the possible different role of cytoplasmic BMP-2 and nBMP-2 in both EMT and microcalcifications production in human breast cancer. To this end, the in-situ expression of BMP-2 and nBMP-2 in human breast cancer was correlated with the expression of the main EMT biomarkers (e-cadherin and vimentin) and molecules involved in bone metabolisms (RUNX2, RANKL, SDF-1) in a cohort of 150 patients.

As expected, both variants of BMP-2 were more expressed in the malignant lesions than the benign ones. Indeed, it is known that EMT, the presence of BOLCs, and breast microcalcifications made of HA or Mg–HAp are associated with breast cancer rather than benignant lesions [12,13,14,15,16,22].

A great difference was found when the association between BMP-2 variants and the main EMT biomarkers in the breast, such as vimentin and e-cadherin, was analyzed. Vimentin is an intermediate filament generally expressed by cells of mesenchymal origin [23]. In breast cancer, the presence of numerous vimentin breast cancer cells is detectable in poor differentiated carcinomas with negative prognosis [24]. Despite some controversial data, the loss of e-cadherin is considered a hallmark of EMT in breast cancer, especially in lobular carcinomas [25]. Remarkably, our data showed a very strong association among the number of cytoplasmic BMP-2-positive cells and the number of both vimentin and e-cadherin-positive breast cells regardless of the breast lesions type. Conversely, no associations were found comparing the number of nBMP-2-positive breast cells and studied EMT markers. Thus, we can speculate that the nuclear variant of BMP-2 (nBMP-2) is not involved in the EMT process. In addition, this data agrees with the study of Huang et al. in which authors demonstrated that BMP-2 can promote EMT in breast cancer via phosphatidylinositol 3-kinases/protein kinase B pathway (PI3K/Akt) [5], a cellular signaling that can be regulated both at extracellular and cytoplasmatic level. The evidence that PI3K/Akt signaling pathway mediates the process of EMT has attracted widespread attention as a potential target for the prevention and treatment of metastatic tumors [26]. In this context, extracellular/cytoplasmic expression of BMP-2 could represent a target for future therapies.

The opposite result was obtained by analyzing the variants of BMP-2 and both the expression of BOLCs biomarkers (RUNX2, RANKL, and SDF-1) and the presence/elemental composition of microcalcifications. Specifically, the presence of microcalcifications made of HA and Mg–HAp, calcifications actively produced by BOLCs [22], were related to the expression of nBMP-2 variant rather than the cytoplasmic one. Concordantly, a strong association between the number of nBMP-2 and the expression of the main BOLCs biomarkers was found. In particular, these three biomarkers are physiologically involved in both osteoblast differentiation and activities (the production of calcifications). RUNX2 is an essential transcription factor; during osteoblast differentiation, RUNX2 is weakly expressed in uncommitted mesenchymal cells, and its expression is upregulated in pre-osteoblasts, reaches the maximal level in immature osteoblasts, and is down-regulated in mature osteoblasts [27]. In breast cancer, the expression of RUNX2 has been related to both BOLCs [28] and cancer metastatization [29]. Considering this information, we are not surprised to note that the number of RUNX2-positive breast cells was associated with both variants of BMP-2. Indeed, the development of BOLCs, rather than their activities, required the EMT phenomenon. On the contrary, our data showed a strong association between the number of nBMP-2-positive breast cells and RANKL, a molecule expressed in mature osteoblasts capable of producing HA crystals [30]. No association was found considering the cytoplasmatic expression of BMP-2. In addition, the same result was observed for the expression of SDF-1. This is a chemokine signaling molecule involved in osteoblast differentiation in both in-bone and extra-bone sites, where it can induce the formation of ectopic calcifications [31]. In breast cancer, the over-expression of SDF1 protein was associated with positive estrogen receptor (ER) status, the breast cancer subtype previously associated with the presence of BOLCs and the development of bone metastases [28,32]. The investigation concerning the microcalcifications, as well as the expression of RANKL and SDF-1, support the idea that nBMP-2 is involved in BOLCs differentiation and activities rather than EMT phenomenon. Ex vivo data obtained on bioptic human tissues were further confirmed by in vitro investigations. Specifically, according to our recent study [12], we developed an in vitro experiment in which BT-474 cells were cultured with CO in order to induce their differentiation into osteoblast-like cells. In these conditions, BT-474 cells showed simultaneous expression of nBMP-2 and both RANKL and SDF-1. In both controls (BT-474 cells alone) and in the presence of HA, only rare cells displayed these immunophenotipical characteristics (the simultaneous expression of nBMP-2 and RANKL). This support the idea that nBMP-2 is involved in BOLCs and microcalcifications origin.

## 5. Conclusions

The identification of cellular and molecular mechanisms involved in EMT phenomenon, BOLCs origin, and microcalcifications production can lay the foundation for identifying both new prognostic biomarkers and therapeutic procedures. Indeed, the identification of molecular events linked to these biological phenomena could highlight new molecular targets for breast cancer therapy and prevention. In this context, the inhibitors of BMP signaling have been proposed as a possible therapy for several diseases including cancer [33]. Also, in some research protocols, BMPs or BMPs inhibitors have been marked with radioisotopes to study in real-time in vivo, by PET or SPECT, their effect on cancer progression. In the future, these protocols could be used to develop diagnostic investigations based on nuclear medicine studies. In our previous study, we used similar approaches to find possible in vivo imaging markers to discriminate the breast cancer characterized by the presence of BOLCs and microcalcifications to those without them [34,35].

In conclusion, from these experimental and translational perspectives, the complexity of BMP-2 signaling will require a detailed understanding of the involvement of specific BMP-2 variants in breast cancers.

## Figures and Tables

**Table 1:** List of primary antibodies.

Antibody	Characteristics	Dilution	Retrieval
Anti-BMP2	Mouse monoclonal clone 1A11; Novus Biologicals, Littleton, CO, USA	1:500	Citrate pH 6.0
Anti-Vimentin	Mouse monoclonal clone V9; Ventana, Tucson, AZ, USA	Pre-diluted	EDTA citrate pH 7.8
Anti-E-cadherin	Mouse monoclonal clone (36); Ventana, Tucson, AZ, USA	Pre-diluted	EDTA citrate pH 7.8
Anti-RUNX2	Mouse monoclonal clone 3F5; Novus Biologicals, Littleton, CO, USA	1:100	Citrate pH 6.0
Anti-RANKL	Rabbit monoclonal clone 12A668; AbCam, Cambridge, UK	1:100	EDTA citrate pH 7.8
anti-SDF-1	Mouse monoclonal clone 79018; Novus Biologicals, Littleton, CO, USA	1:100	EDTA citrate pH 7.8

## Abbreviations

BMPs	Bone morphogenetics proteins
BMP-2	Bone morphogenetic protein-2
BMP-4	Bone morphogenetic protein-4
BOLCs	Breast osteoblast-like cells
CO	Calcium oxalate
EDX	Energy dispersive X-ray
EMT	Epithelial to mesenchymal transition
ER	Estrogen receptor
H&E	Hematoxylin and eosin
HA	Hydroxyapatite
Mg–Hap	Magnesium-substituted hydroxyapatite
nBMP-2	Nuclear variant of bone morphogenetic protein-2
OPN	Osteopontin
PI3K/Akt	Phosphatidylinositol 3-kinases/protein kinase B
PTX3	Pentraxin-related protein 3
RANKL	Receptor activator of nuclear factor kappa-Β ligand
RUNX2	Runt-related transcription factor 2
SDF-1	Stromal cell-derived factor
TEM	Transmission electron microscopy
